# New reconstruction algorithm for stack artifact reduction in breathing-adaptive four-dimensional computed tomography

**DOI:** 10.1016/j.phro.2026.101102

**Published:** 2026-10-03

**Authors:** Juliane Szkitsak, Maximilian Heider, Niklas Andre Lackner, Charlotte Frei, Allison Lamrani, Christopher Dürrbeck, Annika Oleszczuk, Mushawar Ahmad, Jannis Dickmann, Janis Langkrär, Oliver J. Ott, Stefanie Corradini, Rainer Fietkau, Christoph Bert

**Affiliations:** a Department of Radiation Oncology, Universitätsklinikum Erlangen, Friedrich-Alexander-Universität Erlangen-Nürnberg, Erlangen, Germany; b Comprehensive Cancer Center Erlangen-EMN (CCC ER-EMN), Erlangen, Germany; c Bavarian Cancer Research Center (BZKF), Erlangen, Germany; d Siemens Healthineers AG, 91301 Forchheim, Germany

**Keywords:** 4DCT, Stack Artifact Correction, irregular breathing, i4DCT

## Abstract

Background and Purpose: Even with new adaptive four-dimensional computed tomography (4DCT) techniques, artifacts can still occur with strong irregular breathing amplitudes. A new image reconstruction algorithm, currently available for cardiac CT, was tested on 4DCT data for its performance to reduce stack artifacts and thus improve anatomical correctness.

Materials and Methods: The ‘ZeeFree’ algorithm employs a deformable image registration during reconstruction to reduce remaining stack artifacts. Its performance was evaluated using data of 20 irregular breathing (‘challenging group’) and 10 randomly selected (‘verification group’) patients with tumors in the thorax. Using a 5-score-scale, image quality in terms of stack artifacts was rated by three physicians and three physicists. Statistical significance was assessed using the Wilcoxon signed-rank test for the mean ratings and the Chi-square test for anatomical correctness.

Results: Expert scoring revealed that 88.3% of the amplitude-based ZeeFree reconstructions showed (almost) no artifacts (rated with ≥ 4) compared to 66.8% of the standard reconstruction. With phase-based binning, 70.0% of the ZeeFree and 23.3% of standard were rated with a score ≥ 4. Mean scores were 4.1 and 4.0 for amplitude- and phase-based binning with ZeeFree and 3.4 and 2.5 for standard reconstruction, respectively. Differences were significant (*p* < 0.001). 91.1% (ZeeFree) and 41.1% (standard) were rated as anatomically correct. Again, differences were significant (*p* < 0.001).

Conclusions: This study demonstrated a statistically significant reduction of stack artifacts, resulting in improved anatomical correctness when ZeeFree was applied to 4DCTs. The algorithm was not observed to introduce new artifacts on already artifact-free images.

## Highlights

• Four-dimensional computed tomography reconstruction improved images without extra dose.
• No negative influence on already artifact-free images when ‘ZeeFree’ was applied.
• Improvement in radiotherapy due to the reduction of stack artifacts can be expected.
• No or negligible artifacts in 88.3 % of the amplitude-based ZeeFree reconstructions.
• All ZeeFree reconstructions were assessed; 91.1% were anatomically correct.

## Graphical abstract

**Figure:**
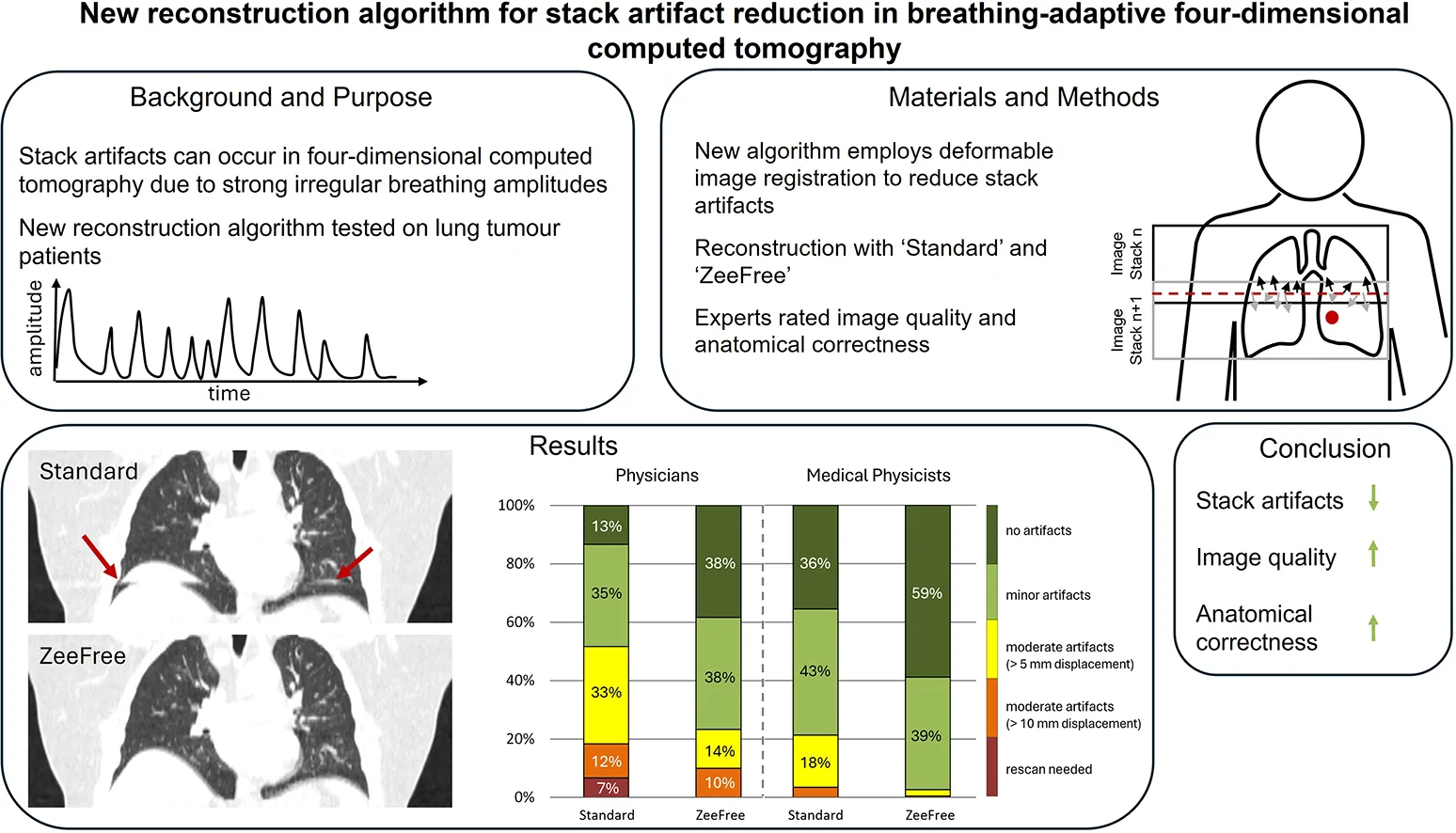

## 1 Introduction

Four-dimensional computed tomography (4DCT) is an integral component in radiotherapy treatment planning, although the technology suffers from artifacts once breathing irregularities occur. As a result, patients' outcome is directly influenced [1]. Modern techniques that adapt data acquisition individually to the patient-specific breathing pattern can largely eliminate artifacts without increasing imaging dose [2], [3].

However, artifacts can still occur when substantial breathing irregularities are present [4]. A common consequence is to repeat (parts of) the 4DCT scan, resulting in unnecessary dose exposure [5], [6]. Several studies have focused on the possibilities of image post-processing to improve quality by reducing artifacts. Deformable and geodesic density registrations as well as deep-learning approaches have demonstrated their potential to effectively reduce motion artifacts [7], [8], [9], [10]. Another option is an optimized setting of binning points during the reconstruction to compensate for missing information [11].

In coronary computed tomography angiography (CCTA), stack artifacts may occur when detector coverage is insufficient to cover the whole heart, affecting up to 18% of scans [12]. In previously published studies, a new reconstruction algorithm showed great potential for reducing stack artifacts and thus increasing image quality [13], [14].

This study evaluated the potential of this reconstruction algorithm to improve image quality for breathing-controlled 4DCTs in radiotherapy. It was analyzed whether the algorithm could significantly increase the number of artifact-free images and anatomical correctness of 4DCT images compared to standard reconstructions.

## 2 Materials and methods

### 2.1 Reconstruction algorithm ‘ZeeFree’

‘ZeeFree’ (Siemens Healthineers, Forchheim, Germany) is a new commercially available reconstruction algorithm aimed to minimize stack artifacts caused by heartbeat or irregular breathing amplitudes in time-resolved CT acquisitions. The reconstruction algorithm was first described by Moser et al. [13] and later by Lisi et al. [14] in application to cardiac CT. According to Moser et al. [13] the algorithm can be summarized in three basic steps: reconstruction using fixed vendor-defined reconstruction parameters independent of user-selected settings, calculation of a vector field that corrects for stack artifacts, application of the vector field to a reconstruction with the user-selected parameters.

Firstly, a weighted filtered back projection is performed to reconstruct an individual cardiac or, in this case, respiratory phase image for each cardiac or respiratory cycle, respectively. To ensure independence from user-specific settings (e.g. kernel, matrix size, etc.), fixed vendor-defined reconstruction parameters are used. This results in a set of reconstructions, with each having a coverage in the *z*-direction equivalent to a single full detector collimation (stack; approx. 4–6 cm depending on the CT scanner). Two neighboring stacks will have an overlap of approximately 10%.

This overlap is exploited in a second step, where at the midplane of each overlap area, a 3D vector field is derived using a DEMON type deformable registration algorithm. The registration brings the overlapping stacks to agreement at the midplane using a joint 3D vector field with opposite signs for the two stacks, allowing non-rigid displacements in all three spatial directions.

To obtain a full-volume field in the final step, all individually obtained 3D vector fields (one at each midplane) are distance-weighted, interpolated and resampled. The resulting vector field is then defined across the entire image volume and applied during the reconstruction with user-selected reconstruction parameters (e.g. slice thickness, kernel, iterative reconstructions, etc.). Details about the algorithm, its technical background and the mathematical principles underlying this type of solution can be found in previous publications [13], [14], [15] and in the white paper [16].

### 2.2 Patient cohort

Breathing curves from 465 clinical 4DCT scans of patients with tumors in the thorax (acquired from 01/2020 to 11/2021) were analyzed with regard to their amplitude variations. The relative standard deviation of the peak-to-valley amplitude of their average respiratory cycle (relative amplitude variation) was determined. The 20 most variable curves and their corresponding raw data were selected (‘challenging group’). To verify that the algorithm had no negative impact on images with no or only minor artifacts, a second cohort was also selected for verification (‘verification group’). For this purpose, ten patients (acquired from 01/2024 to 03/2024) were randomly selected without knowledge of the severity of their respiratory irregularities. Details of both patient cohorts can be found in Table 1.

**Table 1:** Patient details for the challenging and verification group. Patients received either normo-fractionated or stereotactic radiotherapy. The diagnosis ‘Other’ includes metastases in the lymph nodes or ribs. Relative amplitude variability describes the relative standard deviation of the peak-to-valley amplitude of their average respiratory cycle. SCLC = small cell lung cancer; NSCLC = non-small cell lung cancer.

Characteristics	Challenging Group	Verification Group
	*n* = 20	*n* = 10
Sex		
Female	4 (20%)	3 (30%)
Male	16 (80%)	7 (70%)
Age [years]		
Range	47–85	43–84
Median	70	66
Relative amplitude variability [%]	48.46 ± 13.84	18.62 ± 7.03
Range	34.06–107.13	7.65–30.29
Diagnosis		
SCLC	2 (10%)	1 (10%)
NSCLC	6 (30%)	4 (40%)
Lung metastasis	10 (50%)	2 (20%)
Other	2 (10%)	3 (30%)

All patient data used in this study were assessed as part of clinical routine and analyzed retrospectively. All procedures were conducted in accordance with the ethical standards of the institutional research committee and with the Declaration of Helsinki from 1964 and its subsequent amendments.

### 2.3 Acquisition and reconstruction parameters

All patient data were acquired using the i4DCT algorithm [17] implemented in the Direct i4D scan mode on the SOMATOM go.Open Pro scanner (Siemens Healthineers, Forchheim, Germany) in combination with the Respiratory Gating for Scanner System (RGSC, Varian Medical Systems, Inc., Palo Alto, USA). Due to the acquisition using clinical scan protocols, all data had the same acquisition and reconstruction parameters (see reference [4] for details). The reconstruction was performed as a multiphase image (i.e., ten 3DCT images in different respiratory phases) with 3 mm slice thickness and a qualitative kernel Qr40. Each patient data set was retrospectively reconstructed using the new ZeeFree and the standard algorithm with amplitude-based (AB) and phase-based (PB) binning.

### 2.4 Image quality and artifact reduction analysis

To evaluate the potential of ZeeFree, the study investigated whether the algorithm can significantly increase the number of (almost) artifact-free images compared to standard reconstructions, while maintaining or increasing the fraction of anatomically correct 4DCT images. Furthermore, it was analyzed whether the application would have a negative impact on already artifact-free images. To assess image quality, all images were presented in a blinded and randomized order to expert readers in an in-house developed 4DCT viewer. The viewer presented the data by default in the lung window (C = -600 HU, W = 1200 HU). Experts could view all ten phases of a single 4DCT image as a moving animation, scroll through slices in all planes, zoom in and out, and measure distances. Three board-certified physicians and three medical physicists, all with experience > 5 years, served as experts.

Image quality regarding artifact strength was rated on a 5-score Likert scale from 5–no visible artifacts to 1–severe artifacts that necessitate a rescan. Images with no or minimal artifacts, which could be used clinically without restrictions, were scored as 5 or 4. Minor artifacts leading to minor limitations in clinical usability were rated with a 3. Images that could not be used clinically due to severe artifacts or only with severe limitations were rated with a 1 or 2. Details and examples of the individual ratings can be found in the Supplementary Material A.

To assess anatomical correctness, physicians were asked to evaluate on a binary scale whether clear anatomical inaccuracies could be observed, including non-physiological or unnatural distortions, patient surfaces, or movements caused by breathing. In addition, scores < 3 on the Likert scale were automatically considered incorrect, as the anatomy was severely altered by the artifacts.

Standard and ZeeFree reconstructions of the 30 patients were evaluated by two expert groups at different levels of detail. The physicians rated 120 4DCTs (30 patients, both reconstruction algorithms and binning methods) using the Likert scale and anatomical correctness. The medical physicists evaluated only AB-binned 4DCTs, but assessed all ten breathing phases individually, resulting in 30 × 2 × 10 = 600 ratings. The entire volume was evaluated in each case, rather than just individual anatomical regions.

Mean ratings between AB and PB binning method were compared using the Wilcoxon signed-rank test at a significance level of 5%. Significance differences between challenging and verification group were tested with an independent *t*-test. For the statistical analysis of anatomical correctness, Chi-square tests of independence were used.

## 3 Results

For the challenging cases, ‘ZeeFree’ showed a clear advantage over the standard reconstruction for both binning methods. For AB binning, the physicians rated 76.7% (46/60) of the ZeeFree images with a score of 4 and 5 points and therefore (nearly) artifact-free, whereas the standard reconstruction achieved only 48.3% (29/60). The difference was even more profound for PB binning method: 70.0% (42/60) and 23.3% (14/60) were rated with 4 or 5 points for the ZeeFree and standard reconstruction, respectively. The mean scores were 4.1 and 4.0 for AB and PB binning with ZeeFree and 3.4 and 2.5 for standard reconstruction, respectively. None of the ZeeFree, but four of the standard reconstructions using AB binning were rated as 1. All aforementioned differences between ZeeFree and standard were significant (*p* < 0.001). Examples of the differences in image quality for two cases can be found in Fig. 1.

**Fig. 1:**
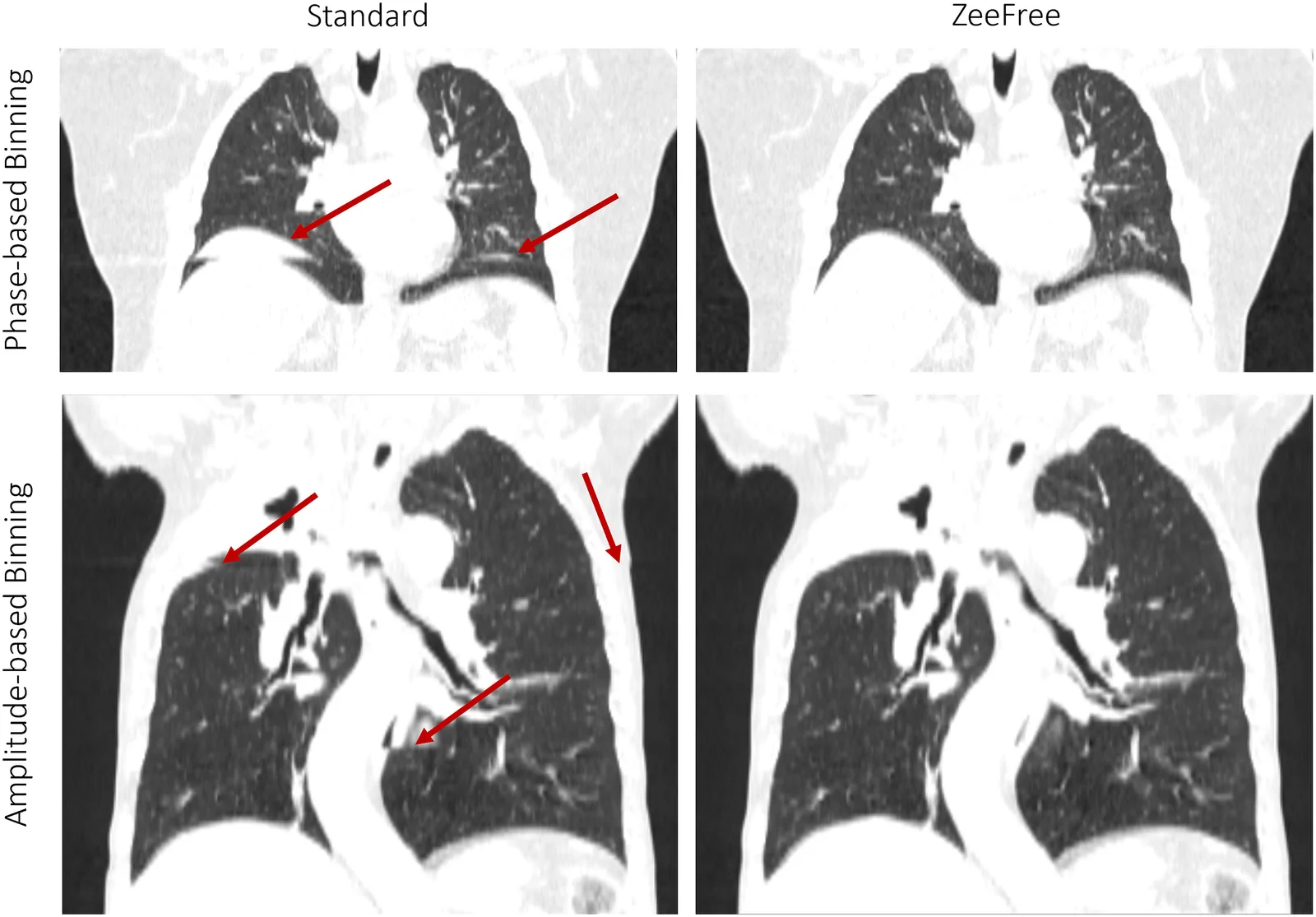
Exemplary cases for image quality of the ZeeFree and standard images with phase-based (top row, patient 9) and amplitude-based (bottom row, patient 11) binning. The red arrows mark stack artifacts resulting from irregular breathing amplitudes. After applying ZeeFree, artifacts can no longer be observed. All images are shown in lung window (Window Center: −600 HU, Window Width: 1200 HU). (For interpretation of the references to colour in this figure legend, the reader is referred to the web version of this article.)

For the physicists, who only assessed AB binning, mean ratings of 4.6 and 4.1 were achieved for ZeeFree and standard reconstruction, respectively. 97.3% (584/600) of the ZeeFree and 78.7% (472/600) of the standard reconstructions received a rating ≥ 4 when considering the challenging cases. Only 13/600 (2.7%) phases were rated with 3 and 3/600 (0.5%) were rated with 2 for the ZeeFree reconstruction. For the standard reconstruction, 107/600 (17.8%) were rated with 3 and 21/600 (3.5%) with 2. Fig. 2 shows the ratings for both expert groups.

**Fig. 2:**
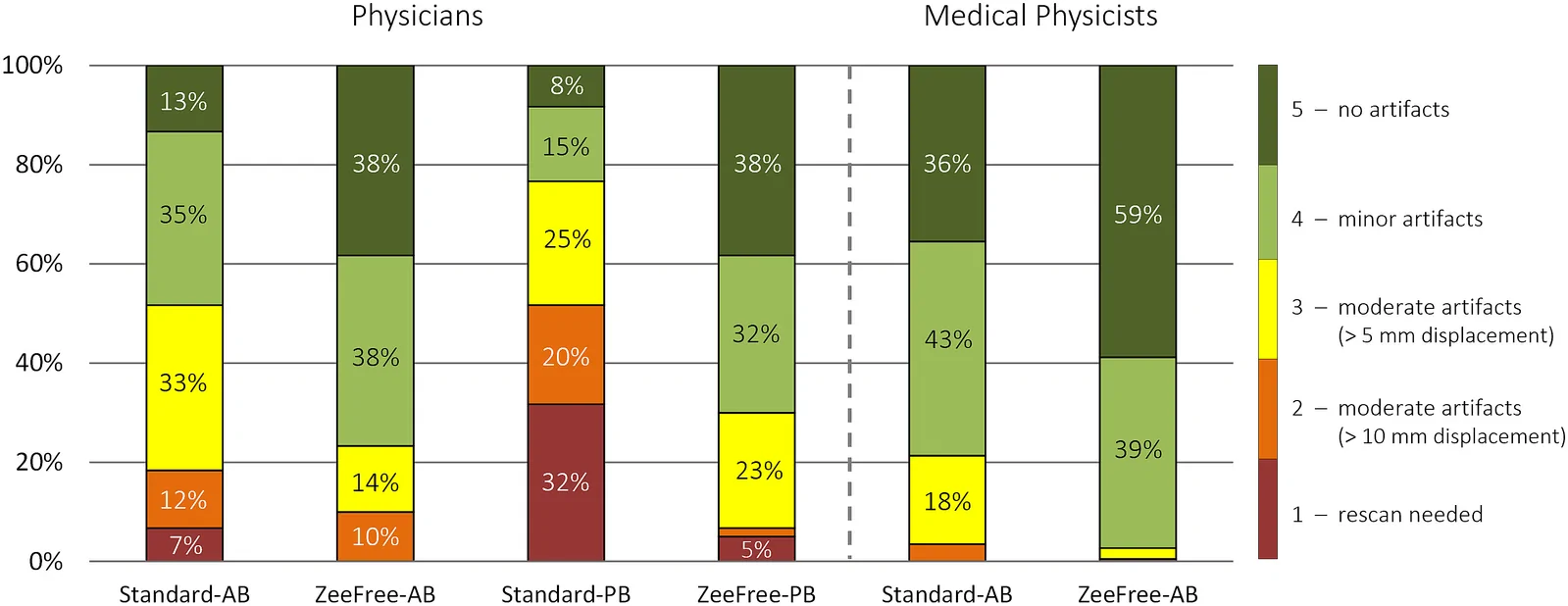
Distribution of ratings for the ZeeFree and standard reconstruction for the challenging cases for (left) the physicians for all 4DCTs reconstructed amplitude- (AB) and phase-based (PB), and (right) the medical physicists for all breathing phases with amplitude-based binning.

The verification group aimed to investigate whether the algorithm had a negative impact on image quality if artifacts were absent or less pronounced. The mean ratings of the physicists were 4.4 and 4.2 for ZeeFree and the standard reconstruction, respectively. Among the physicians, a mean rating of 4.3 was obtained for ZeeFree using both AB and PB binning for the verification group. For standard reconstruction, the mean scores were 3.4 and 3.2. The observed differences between the reconstruction algorithms were significant (*p* < 0.001). 56.7% (17/30) of ZeeFree ratings of the physicians were rated with 5 for AB and PB binning for the verification group. In comparison, only 20.0% (6/30) and 16.7% (5/30) achieved a score of 5 for standard reconstruction within the verification group. Significant differences between the results for the physicians of the challenging and verification group were observed only for the PB binning standard reconstruction (*p* < 0.05) with a mean rating of 2.5 and 3.2, respectively. For the ZeeFree as well as the AB binning standard reconstruction, no significant difference could be observed (*p* > 0.05 for all other reconstruction combinations). For the medical physicists a significant difference was only observed for the ZeeFree algorithm (*p* < 0.001) with mean ratings of 4.6 and 4.4 for the challenging and verification group, respectively.

Fig. 3 shows a detailed comparison of the average ratings of the physicians for the individual scans for both patient groups. For the verification and challenging group as well as for AB and PB binning methods an improvement could be achieved by using ZeeFree. Only for patient 4 and patient 20, a decrease in the mean rating was observed. A closer look at these two patients revealed, that two physicians assigned the same rating, while the third physician gave a rating one point lower. This indicates that no severe degradation was caused by the algorithm and may also be due to the uncertainty of the used Likert scale. Fig. S1 shows the same detailed overview for the evaluations of the physicists.

**Fig. 3:**
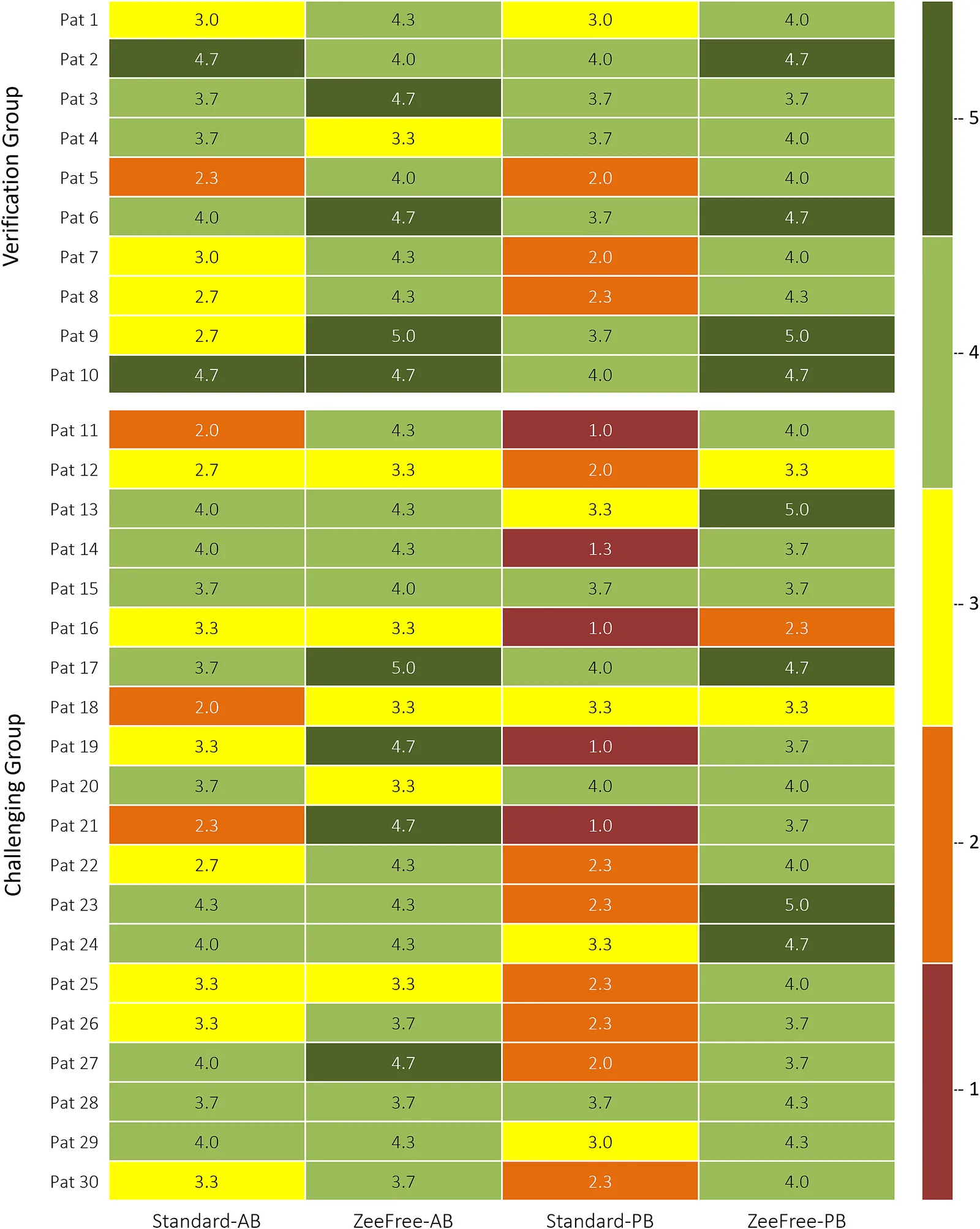
Average physician ratings for amplitude-based (AB) and phase-based (PB) binning applying ZeeFree and standard reconstruction in the verification and challenging groups. Artifact strength was rated on a 5-score Likert scale from 5–no visible artifacts to 1–severe artifacts that necessitate a rescan. Minor artifacts leading to minor limitations in clinical usability were rated with a 3.

The transition matrices of both expert groups revealed further information about the behavior of the stack artifacts and thus the image quality when using ZeeFree (Fig. 4). For the physicians, the respective images of the standard and ZeeFree algorithms were compared regardless of binning method. For the physicists, all individual phases of the 4DCTs were considered. The matrices include the verification as well as the challenging group. For 40.6% of the physicians' and 19.3% of the physicists' cases initially rated with ≤ 3 with standard reconstruction, ZeeFree resulted in (almost) no artifacts (rating ≥ 4). In contrast, only 2.3% / 2.4% of images rated by physicians / physicists as (almost) artifact-free with standard reconstruction (rating ≥ 4) showed artifacts (rating ≤ 3) with ZeeFree reconstruction. It must be taken into account, that differences between consecutive ratings were small and therefore a one-point difference in rating could occur easily. Rating difference ≥ 2 points are more meaningful, as they are more likely to indicate a substantive difference between the reconstruction methods. An increase of ≥ 2 was observed for 36.1% and 11.9% of the ratings by the physicians and physicists, respectively. 35.0% and 48.4% of the images evaluated by the physicians and physicists, respectively, showed no differences between ZeeFree and standard reconstruction.

**Fig. 4:**
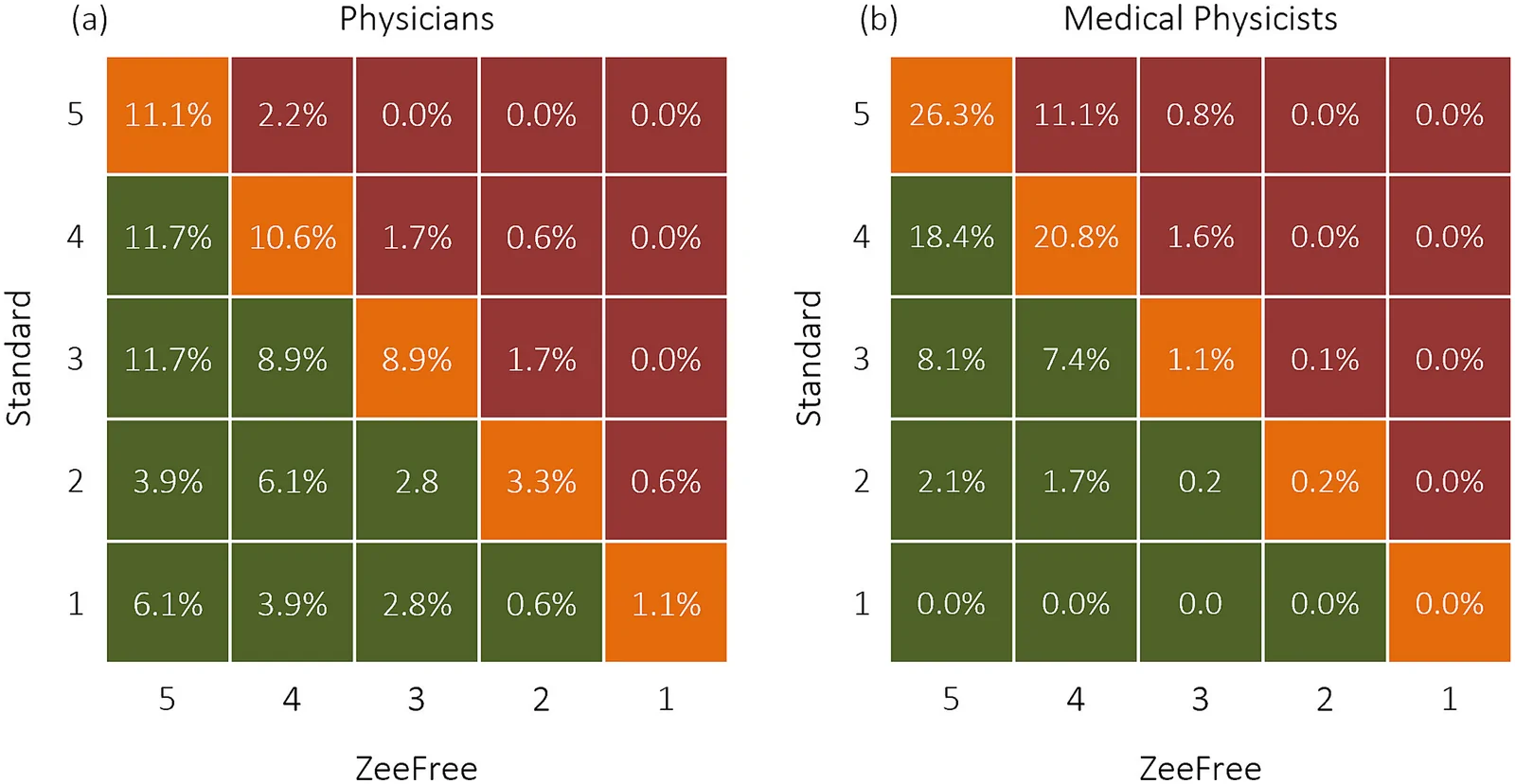
Transition matrix between standard and ZeeFree reconstruction for (a) physicians and (b) medical physicist. The transition matrix for the physicians includes both amplitude- and phase-based binning for the entire 4DCT image. The medical physicists transition matrix contains only amplitude-based binning but for all ten phases of the 4DCT image. A decrease in rating is highlighted in red, an increase in green and a constant rating in orange. (For interpretation of the references to colour in this figure legend, the reader is referred to the web version of this article.)

The analysis of the anatomical correctness revealed significant differences between ZeeFree and standard reconstruction. For all physicians' ratings, only 16/180 (8.9%) of the ZeeFree 4DCTs were rated as anatomically incorrect compared to 74/180 (41.1%) of the standard reconstructions. Accordingly, 91.1% (ZeeFree) and 58.9% (standard) were rated as anatomically correct. The Chi-square test of independence yielded a highly significant *p*-value of ≤ 0.001, indicating a strong correlation between chosen algorithm and anatomical correctness. The transition matrix, containing AB and PB binning for the physicians, clearly demonstrated that ZeeFree resulted in consistent or improved anatomical correctness (Fig. 5). A closer look at the binning methods revealed that for AB 91.1% (82/90) and 73.3% (66/90) for the ZeeFree and standard reconstruction, respectively, were rated as anatomically correct (*p* < 0.001). For PB binning, ZeeFree achieved similar results. For standard 55.6% (50/90) of the cases were rated as anatomically incorrect. (*p* < 0.001).

**Fig. 5:**
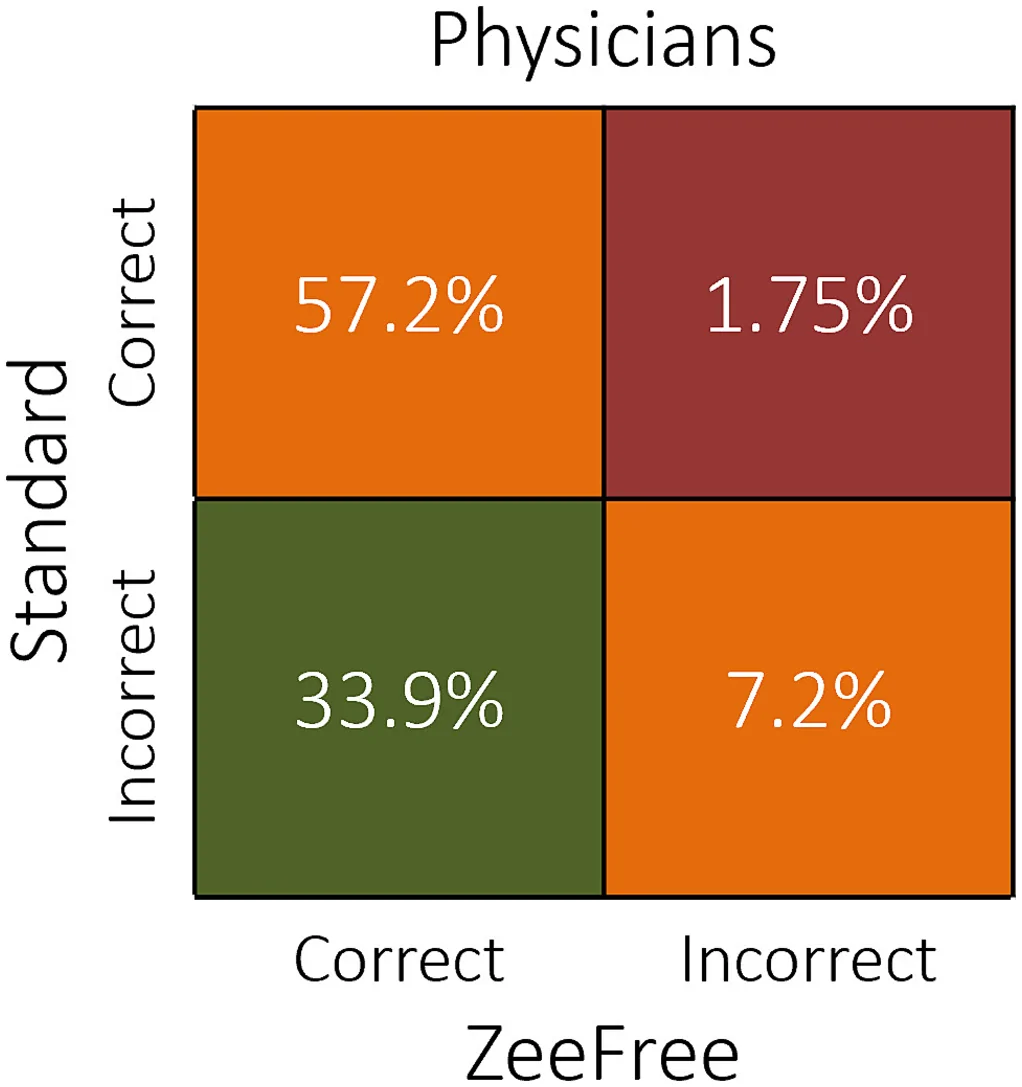
Transition matrix for the evaluation of the anatomical correctness for the physicians. A decrease is highlighted in red, an increase in green and a constant rating in orange. (For interpretation of the references to colour in this figure legend, the reader is referred to the web version of this article.)

## 4 Discussion

This study evaluated the performance of a novel reconstruction algorithm for reducing stack artifacts caused by irregular breathing amplitudes. ZeeFree significantly reduced residual artifacts in i4DCT datasets and improved anatomical correctness while maintaining image quality in artifact-free regions. These findings demonstrate the potential of ZeeFree to further enhance image quality in challenging 4DCT acquisitions.

The algorithm was originally developed for cardiac CT providing promising results [13], [14]. It has now been tested for the first time on clinical thorax 4DCT data. The individual breathing-adaptive acquisition and optimized reconstruction of the i4DCT algorithm have already resulted in significant improvements compared to conventional spiral 4DCT [2], [3], [4], [18]. ZeeFree complements previous improvements in 4DCT by further reducing residual stack artifacts.

The analysis demonstrated, that artifacts caused by breathing amplitude irregularities can be reduced using ZeeFree. The clear improvement of the algorithm over the standard reconstruction, especially for challenging breathing curves with strong amplitude variabilities, strongly supports the results of previous studies in cardiac CT [13], [14]. The advantages were particularly evident with PB binning. One explanation is that PB binning is more prone to artifacts when breathing irregularities occur [3], [4] and therefore has more potential for improvement. A survey among members of the American Association of Physicists in Medicine (AAPM) revealed the need to improve PB binning, as 79% of participants used it for reconstruction in clinical practice [19]. Consistent with previous studies [3], [4], physicists rated image quality higher than physicians. This difference could be attributed to the physicians' larger experience in identifying anatomical inaccuracies, as well as to the different evaluation approaches of the physicians and physicists (overall dataset vs. individual phases).

No significant difference was found between the challenging and the verification group, indicating that the algorithm had no negative impact on already artifact-free images. The analysis of the anatomical correctness showed that 91.1% of the ZeeFree reconstructions were assessed as anatomically correct by the physicians. It must be noted, that a direct comparison with previous studies [3], [4] is not possible. Due to the significant improvement of the i4DCT algorithm through its optimized binning, the breathing-controlled data acquisition and the elimination of interpolation artifacts (previously rated with ≤ 2), the rating scale had to be adjusted to resolve finer differences in the severity of the artifacts. However, this complicates the precise evaluation as the differences between the individual ratings were significantly smaller and thus could easily lead to one-point-differences even with the same artifact characteristic. This was particularly evident in AB binning, which produced fewer artifacts than PB binning. The differences were very subtle, which could easily result in a discrepancy of just one point in the ratings. Additionally, minor artifacts could easily be missed during the evaluation.

One advantage of ZeeFree is avoiding additional dose exposure or postponing therapy start due to a rescan. In the study of the AAPM [19], 5–14% of the participants responded, that 4DCT scans had to be repeated due to artifacts. It demonstrated the still existing influence of motion artifacts on radiation therapy and the need for optimization. However, other reconstruction algorithms rely on the same or a similar principle. Geodesic density or deformable registrations are used to align images of different respiratory phases [7], [8], [9]. Due to their need for intensive computing power and the large data sets, implementation is not easily feasible.

A well-recognized limitation of deformable image registration (DIR) is that multiple deformation fields can produce equally plausible-looking results, making it impossible to identify a unique correct solution [20]. Without a true voxel-level ground truth, systematic errors may therefore remain undetected. No consensus exists on how to define acceptable thresholds to classify DIR performance [21]. In contrast to the broad deformation scenarios commonly addressed in the DIR literature, ZeeFree restricts registration to minor stack-level mismatches within a single acquisition. Lebedev et al. demonstrated in a cardiac CT simulation study with known ground truth that this approach corrected motion-induced stack mismatches while preserving anatomical realism [15]. Consequently, deformation magnitudes are expected to remain small and physiologically plausible, although residual uncertainty cannot be completely avoided.

As with all other methods for reducing motion artifacts, either during data acquisition or image post-processing, the resulting images may suggest more regular breathing than actually occurred during data acquisition. Nevertheless, it is important to take breathing irregularities into account during treatment planning and dose delivery. Respiratory phase gating or tumor tracking can be performed, but the procedures are very complex in the implementation and quality assurance and requires expert knowledge [22], [23]. It would be beneficial if the 4DCT images display the extent of the changes made by the ZeeFree algorithm, for example through visualization of the corresponding deformation vector fields to facilitate assessment of their potential clinical relevance.

In conclusion, the study demonstrated that ZeeFree could achieve a significant improvement in image quality and anatomical correctness when applied to breathing-controlled 4DCTs. A negative influence on already artifact-free images was not be observed.

## CRediT authorship contribution statement

**Juliane Szkitsak:** Writing – review & editing, Writing – original draft, Supervision, Project administration, Methodology, Investigation, Formal analysis, Data curation, Conceptualization. **Maximilian Heider:** Software, Methodology, Investigation, Formal analysis, Conceptualization. **Niklas Andre Lackner:** Writing – review & editing, Investigation, Conceptualization. **Charlotte Frei:** Resources. **Allison Lamrani:** Investigation. **Christopher Dürrbeck:** Investigation. **Annika Oleszczuk:** Investigation. **Mushawar Ahmad:** Writing – review & editing, Formal analysis. **Jannis Dickmann:** Writing – review & editing, Investigation, Conceptualization. **Janis Langkrär:** Investigation, Conceptualization. **Oliver J. Ott:** Writing – review & editing, Resources, Investigation. **Stefanie Corradini:** Resources. **Rainer Fietkau:** Resources. **Christoph Bert:** Writing – review & editing, Supervision, Resources, Methodology, Investigation, Conceptualization.

## Ethics declaration

The authors have NOT obtained informed consent from participants or their legal representatives. All patient data used in this study were assessed as part of clinical routine and analyzed retrospectively. All procedures were conducted in accordance with the ethical standards of the institutional research committee and with the Declaration of Helsinki from 1964 and its subsequent amendments.

This study was performed in compliance with relevant laws, regulatory frameworks and guidelines where the research took place.

Ethics committee approval was not required under relevant laws and institutional guidelines. All patient data used in this study were assessed as part of clinical routine and analyzed retrospectively. All procedures were conducted in accordance with the ethical standards of the institutional research committee and with the Declaration of Helsinki from 1964 and its subsequent amendments.

## Declaration of competing interest

The authors declare the following financial interests/personal relationships which may be considered as potential competing interests: The Universitätsklinikum Erlangen and also the Department of Radiation Oncology have institutional research grants with Siemens Healthcare GmbH.

## Appendix A Supplementary data

Supplementary material: Supplementary data provides the detailed instructions for the rater study incl. example images and a figure with the detailed overview for the evaluations of the physicists.
