# Supplementary material for: New reconstruction algorithm for stack artifact reduction in breathing-adaptive four-dimensional computed tomography
Source: Phys Imaging Radiat Oncol. 2026 Oct 3;41:101102. doi: 10.1016/j.phro.2026.101102 (PMC13675740; doi:10.1016/j.phro.2026.101102)
Supplement: Supplementary file 1 [file mmc1.pdf]

New reconstruction algorithm for stack artifact reduction in breathing-adaptive four-dimensional computed tomography

Supplementary Material A: Instruction for Rater Study

All experts were instructed with the following guidelines:

Rating 5: No recognizable artifacts; clinically usable without restrictions.

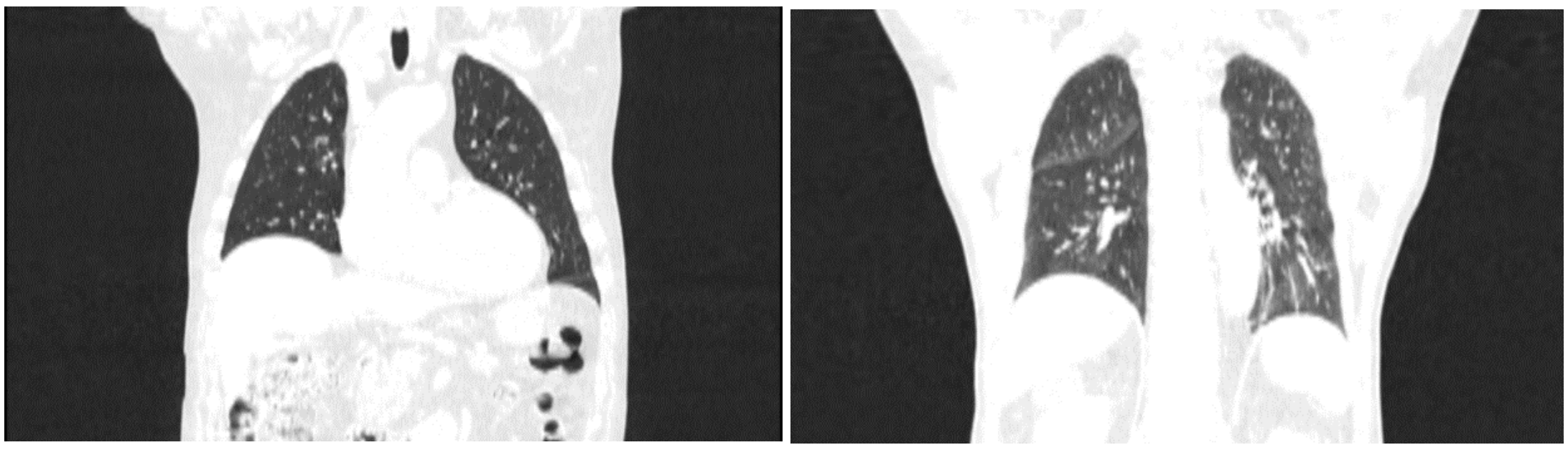

Rating 4: Minimal artifacts; clinically usable without restrictions.

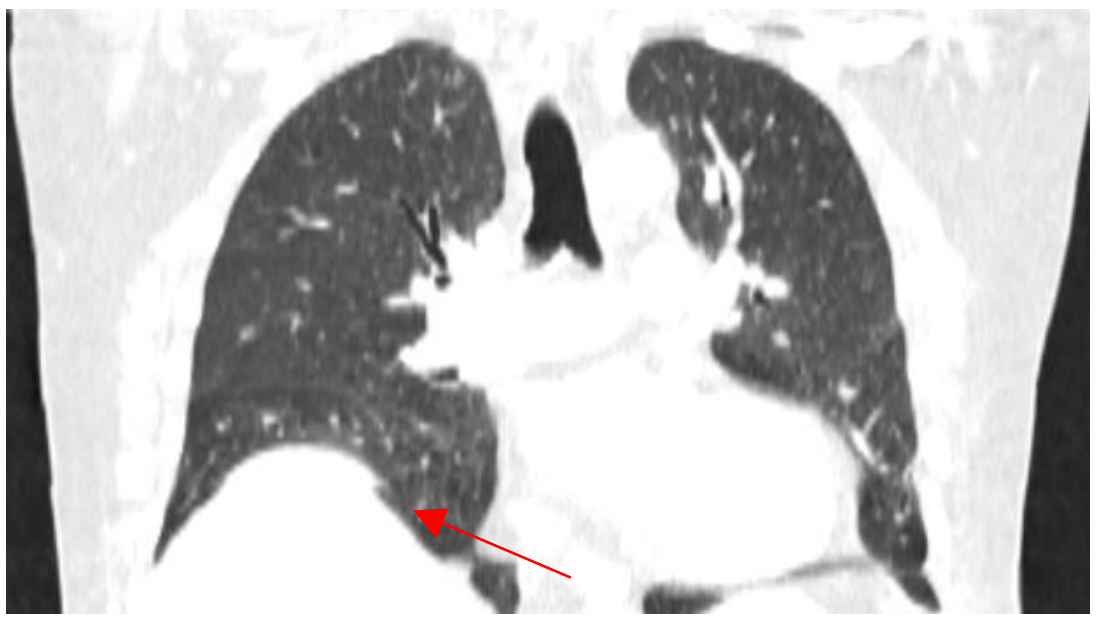

Rating 3: Moderate Artifacts with >5 mm displacement.

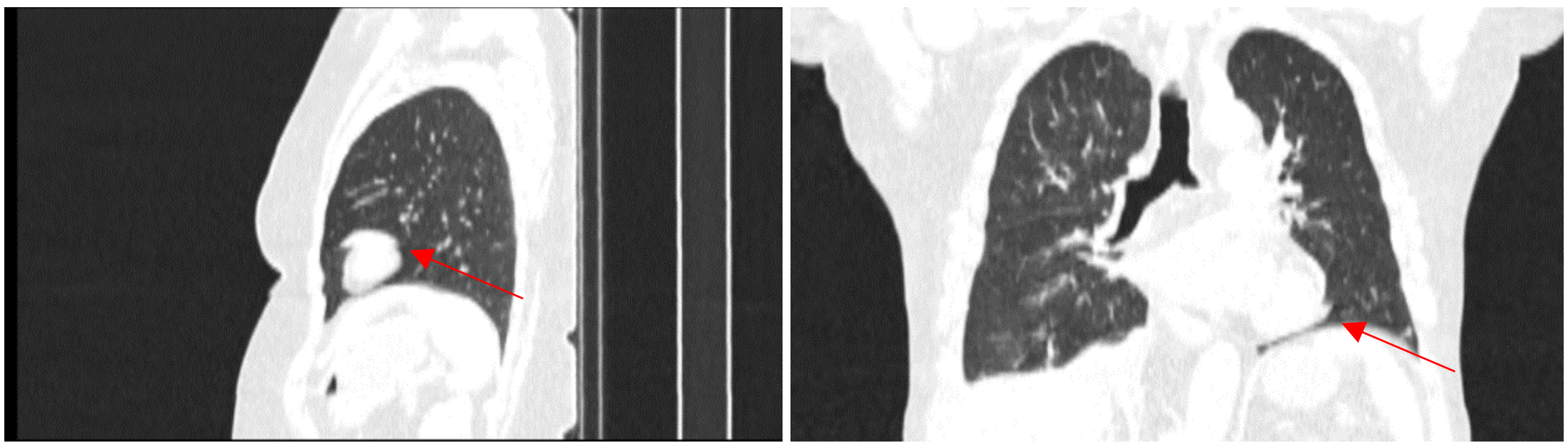

Rating 2: Moderate artifacts with >10 mm displacement.

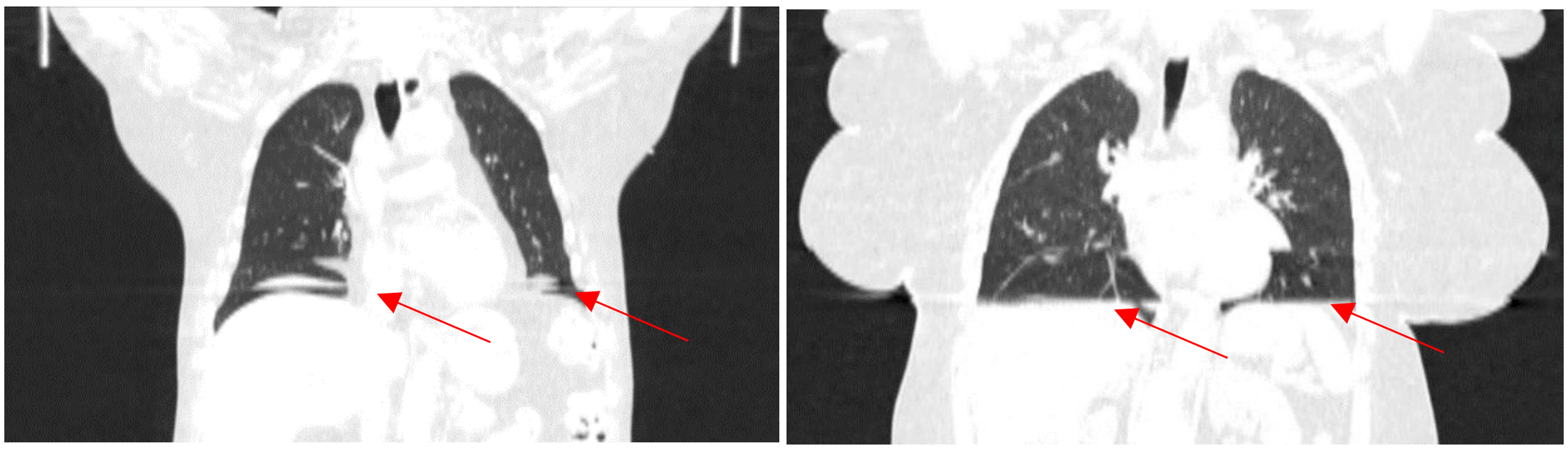

Rating 1: Severe artifacts; rescan required or clinically usable with limitations.

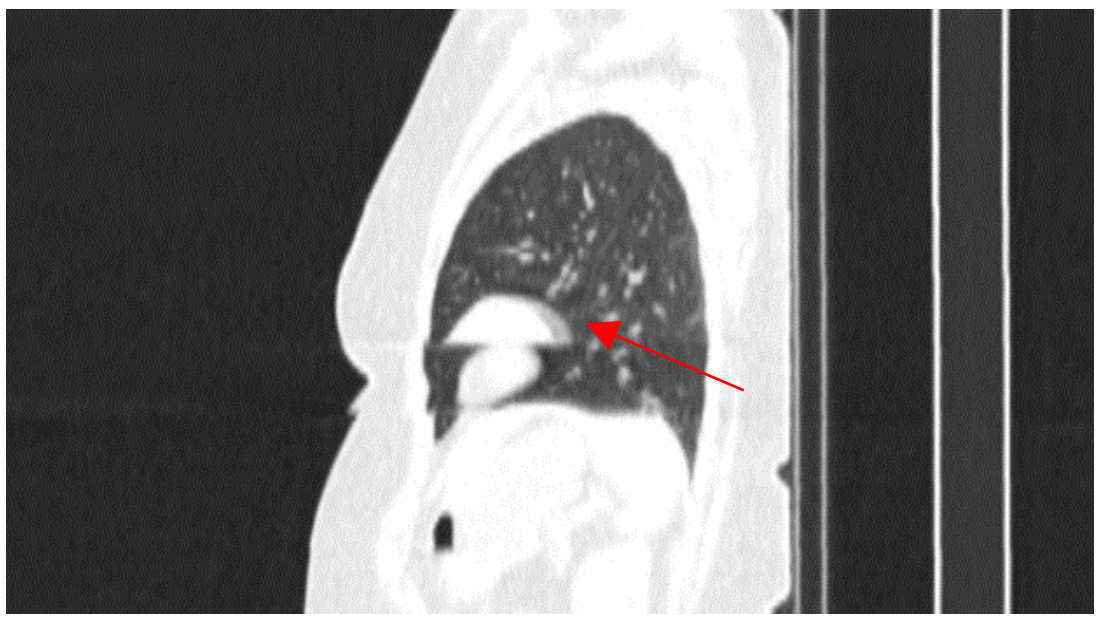

Quality criteria such as noise or contrast should not be taken into account.

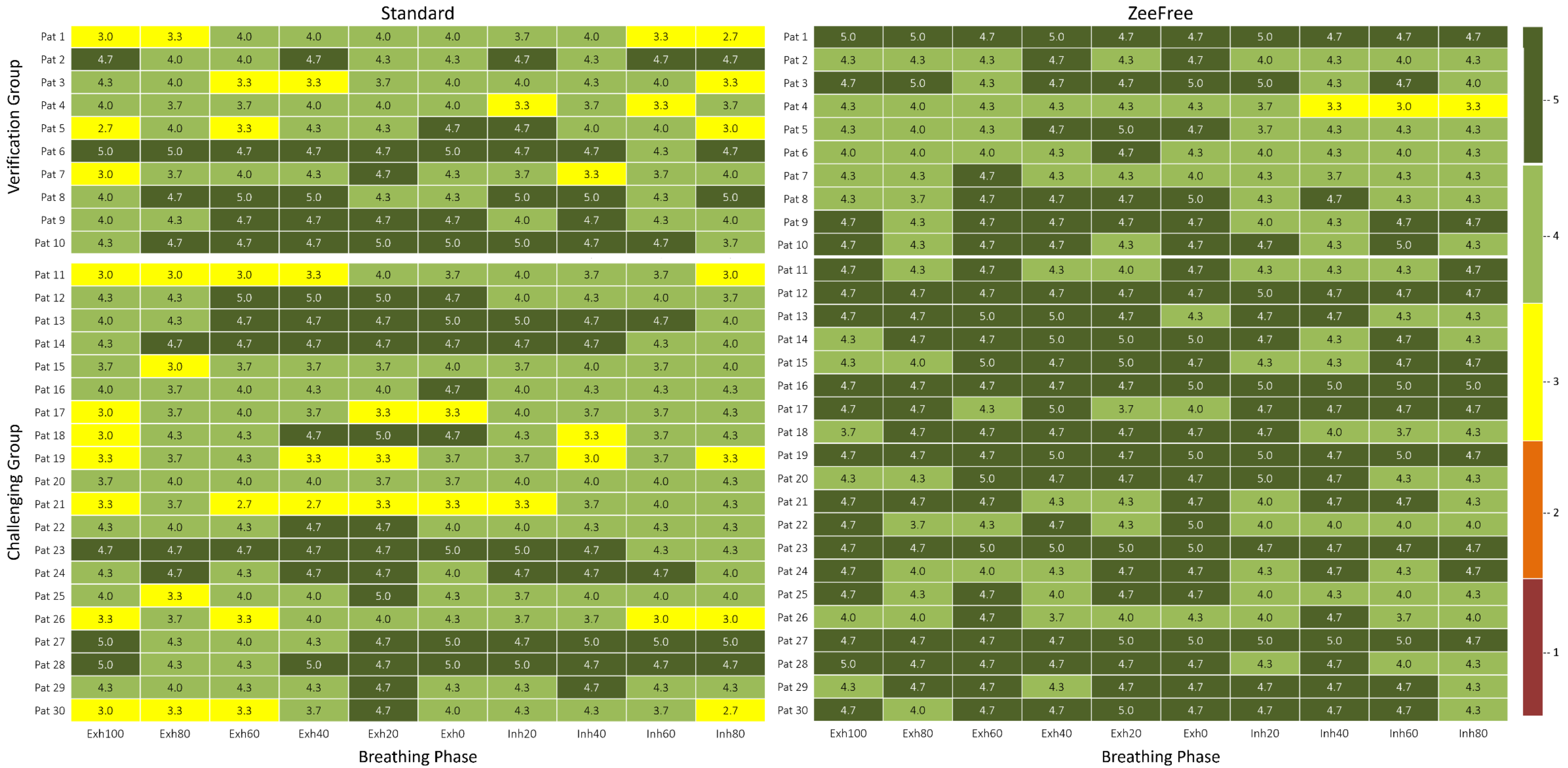

Figure S1: Heatmap of the mean ratings of the medical physicists for all ten breathing phases with amplitude-based binning. Left: mean ratings of all three medical physicists for standard reconstruction for the verification and the challenging group. Right: mean ratings for the ZeeFree reconstructions. The x-axis indicates the breathing phase as inspiration (In) or expiration (Ex), followed by the corresponding percentage of the respiratory amplitude.
